# IRF3 Knockout Results in Partial or Complete Rejection of Murine Mesothelioma

**DOI:** 10.3390/jcm10215196

**Published:** 2021-11-07

**Authors:** Masaya Aoki, Licun Wu, Junichi Murakami, Yidan Zhao, Hana Yun, Marc de Perrot

**Affiliations:** 1 Latner Thoracic Surgery Research Laboratories, Division of Thoracic Surgery, Toronto General Hospital, Princess Margaret Cancer Research Centre, University Health Network, University of Toronto, Toronto, ON M5G 1L7, Canada; mtytah@gmail.com (M.A.); licun.wu@uhnresearch.ca (L.W.); gashan.murakami@gmail.com (J.M.); yidanzhao@gmail.com (Y.Z.); Zhihong.Yun@uhnresearch.ca (H.Y.); 2 Institute of Medical Science, University of Toronto, Toronto, ON M5S 1A8, Canada; 3 Department of Immunology, University of Toronto, Toronto, ON M5S 1A8, Canada

**Keywords:** malignant pleural mesothelioma (MESO), interferon regulatory factor 3 (IRF3), cGAS/STING signaling pathway, IRF3 knockout (IRF3KO), immune checkpoints

## Abstract

Background: Malignant pleural mesothelioma (MESO) has a poor prognosis despite aggressive treatment with surgery, radiation and chemotherapy, and novel therapeutic approaches are needed. IRF3 is a downstream molecule of the cGAS/STING signaling pathway, but its roles have not been investigated in MESO. Methods: Various murine mesothelioma cell lines were inoculated into wild type (WT) and IRF3 knockout (IRF3KO) mice to compare tumor growth. AE17-bearing mice were treated with local radiotherapy (LRT) to evaluate the effect on tumor growth, and immune cell infiltration was analyzed by flow cytometry 20 days after tumor inoculation. TCGA data were used to examine the relationship between mRNA expression of IRF3 and genes of the cGAS/STING signaling cascade on prognosis in MESO. Correlations between gene expression of IRF3, cGAS/STING signaling pathway, and immune checkpoints were analyzed in TCGA MESO and our scRNA-Seq data from MESO patients. Results: In mouse mesothelioma models, AK7, RN5 and ZiP3 were completely rejected in IRF3KO mice 20 days after the tumor challenge. AE17tumor volume was slightly larger than WT mice around day 10 before shrinking and becoming significantly smaller than WT mice on day 20. LRT accelerated tumor shrinkage of AE17 tumors in IRF3KO mice. Compared with WT mice, the number of macrophages infiltrating the tumor of IRF3KO mice was significantly reduced, and CD4^+^ T cells and CD8^+^IFNγ^+^ T cells were significantly increased. TCGA data showed that *IRF3* expression was an unfavorable prognostic factor in MESO patients. *IRF3* expression, the cGAS/STING signaling pathway, and immune checkpoints were positively correlated. Conclusion: *IRF3* could play a critical role in the tumor immune microenvironment of MESO.

## 1. Introduction

Malignant pleural mesothelioma (MESO) is a rare but highly aggressive malignancy. The prognosis is poor even though multimodality treatments including surgery, radiation, and chemotherapy are being performed [1,2]. More recently, our team has achieved encouraging progress by treating resectable MESO with a new approach, Surgery for Mesothelioma After Radiation Therapy (SMART) [3,4,5]. The median survival time (MST) of this approach has shown to be up to 28.3 months, which is better than other clinical phase 2 trials of multimodality treatment for MESO (MST: 16.8–19.9 months) [6,7,8,9,10].

Platinum and pemetrexed have been used as a first-line chemotherapy for unresectable MESO, but the MST with cisplatin and pemetrexed can be as poor as 12.1 months [11]. Recently, the promising efficacy of immune checkpoint inhibitors for MESO patients has been reported [12,13,14]. This has sparked interest in exploring the synergistic effects of immunotherapy with radiation therapy in MESO and other cancers, which has shown promising results by our group and others [15,16,17].

Nowadays, it is widely accepted that both innate and adaptive immunity play important roles in tumor immune surveillance, and that type 1 interferon (IFN) provides an effective antitumor immunity by bridging the innate and adaptive immune system. Recent evidence indicated that cyclic GMP-AMP synthase (cGAS)/stimulator of IFN genes (STING) signaling is important for the induction of type 1 IFN and plays an important role in cancer immunity. The cytoplasmic double strand DNA of cancer cells binds to and activates cGAS, an enzyme that catalyzes the production of cyclic GMP-AMP (cGAMP), which is a type of cyclic dinucleotide that binds to and activates STING [18]. Activated STING changes its conformation to mobilize TANK-binding kinase 1 (TBK1) and phosphorylates IFN regulatory factor 3 (IRF3). Phosphorylated IRF3 translocates to the nucleus and induces the production of type 1 IFN and other cytokines involved in immunomodulation [19]. IRF3 expression has been reported to correlate with prognosis in various carcinomas [20]. A recent study demonstrated that overexpression of IRF3 predicted poor prognosis in clear cell renal cell carcinoma, indicating that IRF3 could serve as promising prognostic marker and therapeutic target in renal cancer [21]. Potential therapeutic targets of STING agonists plus immune checkpoint inhibitor have been reported to achieve treatment benefit in oral squamous cell carcinoma [22]. The cGAS/STING pathway was also shown to be a possible therapeutic target in the inflammatory microenvironment and cancer [23,24].

In this study, we evaluated the tumor growth of various murine mesothelioma cell lines in wild-type (WT) compared to IRF3 knockout (IRF3KO) mice. AE17-bearing mice were treated with local radiotherapy (LRT) to evaluate the effect on tumor growth, and immune cell infiltration was analyzed. The Cancer Genome Atlas (TCGA) data were retrieved to examine the prognostic impact of mRNA expression of *IRF3* as well as genes of the cGAS/STING signaling cascade in MESO patients. Gene expression of *IRF3*, the cGAS/STING signaling pathway and immune checkpoints were analyzed in TCGA MESO and our scRNA-Seq data from MESO patients.

## 2. Materials and Methods

### 2.1. Tumor Cell Lines and Mice

Murine mesothelioma cell line AK7 was provided by Brown University, USA. RN5 and ZiP3 cell lines were developed from C57BL/6 background mice after intraperitoneal injection of asbestos fibers [25,26]. The AE17 cell line was provided by Dr. Steven Albelda, University of Pennsylvania (Philadelphia, PA, USA), and Dr. Delia Nelson (University of Western Australia, Crawley, Australia). All cell lines were grown in RPMI1640 culture media (Life Technologies Inc. Burlington, ON, Canada) supplemented with 10% heat-inactivated FBS (Life Technologies Inc.), 2 mmol/L L-glutamine, 100 U/mL penicillin, 100 mg/mL streptomycin and nonessential amino acids. Cells were plated in tissue-culture coated flasks (BD Biosciences Canada, Mississauga, ON, Canada), grown in a 37 °C and 5% CO_2_ environment, and passaged when 70% confluent. Eight to 12 weeks old C57BL/6 syngeneic mice and IRF3KO mice were initially purchased from Jackson Laboratories, and acclimatized in the animal colony for 1 week before experimentation. The animals were housed in microisolator cages, 5 per cage, in a 12 h light/dark cycle. Sterile water and rodent food were given *ad libitum*.

### 2.2. Mouse Genotyping

DNA was extracted from a piece of tail and/or ear of mice using EZ tissue/Tail PCR genotyping Kit (EZ BioResearch, St. Louis, MO, USA) according to the manufacturer’s instructions. Amplification of *IRF3 mutant* and WT DNA was performed in a total volume of 10 μL, which included 0.5 μL of the DNA product sample, primer (1.5 μL forward primer: 5′-GAACCTCGGAGTTATCCCGAAG-3′, 0.9 μL wild type reverse primer: 5′-GTTTGAGTTATCCCTGCACTTGGG-3′and 0.7 μL IRF3 mutant reverse primer: 5′-TCGTGCTTTACGCTATCGCC-3′), 10 × PCR Buffer –MgCl_2_ (Invitrogen, ThermoFisher, Markham, ON, Canada), 0.4 μL 50 mM MgCl_2_ (Invitrogen), 0.2 U Planium DNA polymerase (Invitrogen) and 0.8 μL 10 mM dNTP (BIO BASIC Canada Inc. Markham, ON, Canada). The amplification reactions were denatured at 94 °C for 3 m, 40 cycles at 94 °C for 20 s, annealing at 59 °C, and elongation at 72 °C for 2 m, and a final elongation at 72 °C for 5 m in the C 1000 Touch Thermal Cycller (Bio-Rad, Mississauga, ON, Canada). Amplified DNA fragments were electrophoresed on 1.5% agarose (BIO BASIC Inc. Montreal, QC, Canada) gel containing Redsafe Nucleic Acid staining solution (IntRON biotechnology, Kirkland, WA, USA) with a DNA Ladder (SMOBiO, DM2300, Mississauga, ON, Canada) for comparison.

### 2.3. In Vivo Tumor Growth Experiments

Mice were subcutaneously injected in the right flank with AK7, RN5, ZIP3 and AE17 (1–2 × 10^6^) cells in 100 μL of PBS. Tumor growth was monitored twice weekly after tumor cell inoculation. Tumor dimensions were measured using a caliper. Tumor volume was calculated according to the formula *V* = (*a* × *b*^2^)/2, where *a* is the largest diameter and *b* is the perpendicular largest diameter. Mice were sacrificed when tumor dimension reached 150 mm^2^ or showed signs of ulceration as per institutional ethics protocols.

We confirm that all animal experiments were carried out in accordance with relevant guidelines and regulations for using animals and approved by University Health Network (UHN) Animal Use Protocol (AUP#3399). Furthermore, we confirm that all the animal studies were carried out in compliance with the ARRIVE guidelines.

### 2.4. Local Radiotherapy (LRT)

Local radiotherapy (LRT) was delivered to the tumor as previously described [27]. AE17 tumors were irradiated using the X-Rad 225Cx small-animal image-guided irradiator (Precision X-ray). The irradiator has a 225 kVp X-ray tube (Varian Associates, Palo Alto, CA, USA) and a flat-panel silicon detector mounted on a 360° rotation C-arm gantry. The automated stage is movable on the *x*-, *y*-, and *z*-axes. It was housed in a self-shielded cabinet and was remotely controlled by a computer (Dell Precision 690, Intel Xeon CPU running Windows XP). The mean targeting displacement error was ≤0.1 mm in the *x*-*y*-*z* planes. Radiation was provided to mice under isoflurane anesthesia. To initially visualize the animal, the tumor fluoroscopic mode was used. To precisely target the tumor, a scout cone-beam computed tomography was created at a 40-kVp tube potential and 0.5-mA current. The tomography was then reconstructed at a 0.4-mm voxel size. The beam source was collimated to either a 1.5- or 2-cm diameter circular field. To confirm the area to be irradiated, the tumor was then visualized under fluoroscopic imaging with the collimator in place, immediately prior to delivery of treatment. Radiation was delivered at a tube potential of 225 kVP and a 13-mA current for a dose rate of 3.02 Gy/minute. The daily dose was administered from two angles, half from above (180°) and half from below (0°). Total dose was administered in divided fractions over 3 days according to treatment protocols. After radiation, mice were placed back in their cages and housing facilities.

### 2.5. Single Cell Preparation from Tumor Tissues

Tumors were removed and placed in 15 mL conical tubes filled with RPMI1640 culture media and stored on ice until further use. Tissue was chopped into 2 mm pieces and transferred to 15 mL conical tubes containing digestion media consisting of RPMI1640, DNAse (Roche 10104159001) and Liberase TM (Roche Diagnostics, Mississauga, ON, Canada). Tubes were placed in a shaking water bath for 30 min and when the pieces were soft and malleable, the solution was filtered and mashed through a 70 µm cell strainer. Cells were then washed with PBS and remaining cells were counted and viability was assessed.

### 2.6. Flow Cytometry

Cells were resuspended in FACS buffer and stained for 30 min at 4 °C with a-CD16/CD32 Fc block (BD, Pharmingen, Franklin Lakes, NJ, USA), and a combination of the following mouse-specific antibodies: CD3, CD4, CD8, CD45, CD68, F4/80, IFN-γ (eBioscience, ThermoFisher Scientific, Mississauga, ON, Canada) as described previously [17]. All samples were then washed twice with FACS buffer and analyzed immediately using a BD LSR II flow cytometer (BD Biosciences, Mississauga, ON, Canada) and FlowJo V10.7 software (FlowJo LLC, Ashland, OR, USA). Tumor samples were pooled and analyzed as a single sample to have sufficient cells.

### 2.7. Patients with Mesothelioma

Five treatment naïve patients confirmed to have a diagnosis of malignant pleural mesothelioma (MESO) had their tumor biopsy tissues and pleural effusion processed freshly for scRNA-seq analysis. These biopsy tissues were collected from patients who were recruited between 5 September 2019 and 16 September 2020. None of them received any type of treatment. The pleural effusion from 3 MESO patients was also analyzed. This study was approved by our institution (REB#19-5858) and all patients signed the consent forms.

### 2.8. Single Cell RNA Sequencing (scRNA-Seq)

Fresh single cells were obtained from peritoneal lavage in mice. Briefly, the peritoneal cavity was exposed and rinsed with 5 mL PBS per mouse to collect the lavage, and tumor spheroids were removed by filtering with a cell strainer (40 µm in diameter). Only single cells were collected for further analysis.

Human pleural effusion or tumor biopsy in patients were processed by Princess Margaret Genomic Centre, University Health Network (UHN), following the standard protocol (www.pmgenomics.ca; accessed on 31 May 2021). Single cells were prepared from pleural effusion of MESO patients, and tumor spheroids were removed similarly as mouse samples. Tumor biopsy tissues were cut into small pieces and tissue dissociation protocol, published previously at the Princess Margaret Genomic Centre, was then followed [28,29].

Loupe Cell Browser v5.0.0 provided by 10× Genomics was used to analyze single cell gene expression in clusters. A reference mouse or human genome (mm10) was selected to set threshold of mitochondrial UMIs as over-expression of mitochondrial genes that could signify poor quality or dying cells.

### 2.9. Analytical Tools

GEPIA2 survival analysis was carried out. The prognostic value of the expression of *MB21D1*, *TMEM173*, *TBK1*, and *IRF3* mRNA in MPM was evaluated using the online database, Gene Expression Profiling Interactive Analysis 2 (GEPIA2) [17]. GEPIA2 is a web-based tool that delivers fast and customizable functionalities based on TCGA and genotype-tissue expression data, which contains the gene expression data and survival information of many cancer types. To analyze the overall survival (OS) of the patients, we set the median expression as the expression threshold to split the patient samples into high expression and low expression groups and used a Kaplan–Meier survival plot with the hazard ratio (HR), a 95% confidence interval (CI), and a log rank test *p* value. GEPIA2 is available at http://gepia2.cancer-pku.cn/ (accessed on 30 January 2021).

Other online analytical tools, such as Loupe Cell browser https://support.10xgenomics.com/single-cell-gene-expression (accessed on 1 February 2021), https://crescent.cloud/ (accessed on 30 June 2021), and The Cancer Genome Atlas (TCGA) https://www.cbioportal.org/ (accessed on 31 January 2021), were employed to identify the IRF3 and cGAS/STING signaling pathway related gene expression in MESO.

### 2.10. Statistical Analysis

Statistical analysis was performed with GraphPad Prism 8 (GraphPad Inc. San Diego, CA, USA). Unpaired two-tailed Student’s *t* test was used to analyze two groups. A *p* value of less than 0.05 was considered statistically significant. Results were presented as mean ± SEM. *, *p* < 0.05; **, *p* < 0.01; ***, *p* < 0.001 in all figures.

## 3. Results

### 3.1. Tumor Growth Curves of Various Murine Mesothelioma Models

**Figure 1:**
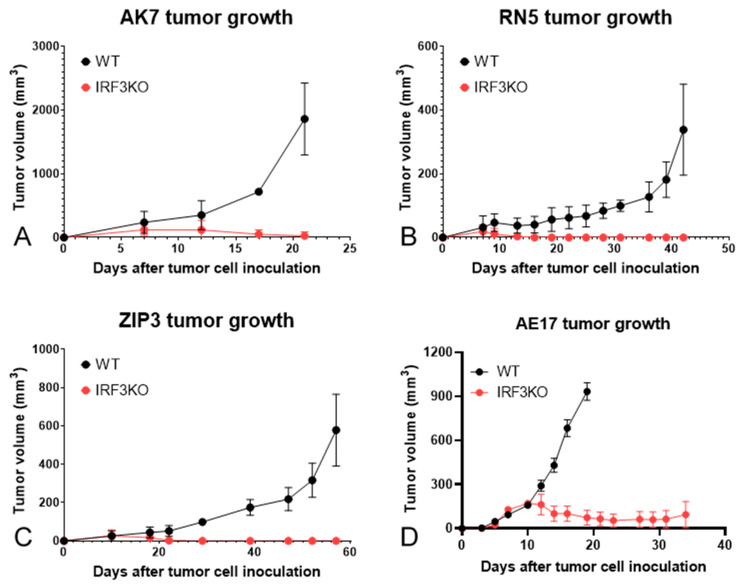
Tumor growth curves of murine mesothelioma in wild-type (WT) vs. IRF3KO mice. Tumor volumes were plotted as a function overtime. (**A**). AK7; (**B**). RN5; (**C**). ZIP3; and (**D**). AE17.

Tumor growth delay was achieved in all mesothelioma models in IRF3KO mice compared with WT mice (Figure 1). Tumors of AK7, RN5, and ZiP3 grew initially and started to shrink around day 10 after tumor cell inoculation, and completely disappeared around day 20 in IRF3KO mice. However, AE17 tumors shrank but did not disappear on day 20 and thereafter. Therefore, LRT was delivered to the AE17 tumor-bearing mice on day 10 in both WT and IRF3KO mice to evaluate the response to LRT.

### 3.2. The Effect of Local Radiotherapy (LRT) on Tumor Growth in WT vs. IRF3KO Mice

**Figure 2:**
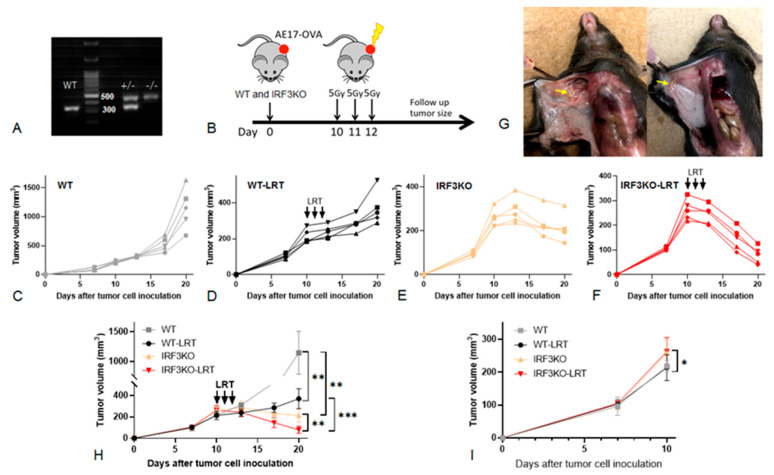
In vivo tumor growth experiments of AE17. (**A**) Genotyping of IRF3KO mice. There were mutant IRF3 heterozygous (IRF3^+/−^) and homozygous mice (IRF3^−/−^). Only IRF3^−/−^ mice were used in our experiments as IRF3KO mice. (**B**) Experimental design. Mice were divided into four groups: WT-LRT group, WT group, IRF3KO-LRT group and IRF3KO group (*n* = 5 per group). LRT was given to WT-LRT and IRF3KO-LRT groups with 5 Gy delivered daily for 3 days (total 15 Gy) from day 10 to 12 after tumor cell inoculation. (**C**) Depiction of the tumor volume in WT group, (**D**) WT-LRT group, (**E**) IRF3KO group, (**F**) IRF3KO-LRT group. (**G**) Image of the tumor in IRF3KO-LRT mice on day 20. (**H**) Tumor growth curves in all groups up to day 20, (**I**) up to day 10; data are mean ± SEM. *, *p* < 0.05; **, *p* < 0.01; ***, *p* < 0.001, unpaired *t* test.

We performed genotyping using the tail and/or ear fragments of IRF3KO mice before starting the experiment. There were mutant IRF3 heterozygous (IRF3^+/−^) and homozygous mice (IRF3^−/−^) (Figure 2A). We used only IRF3^−/−^ mice in our experiments as IRF3KO mice. We previously developed a murine mesothelioma model that mimicked the clinical setting in which mice received non-ablative oligofractionated irradiation with a dose of 15 Gy in three fractions (5 Gy for three consecutive days) [18]. A subcutaneous mesothelioma model was chosen to facilitate the safe delivery of radiation to the tumor. Mice were divided into four groups: WT group without LRT, WT-LRT group, IRF3KO-LRT group and IRF3KO group (*n* = 5 mice per group). LRT was given to WT-LRT and IRF3KO-LRT in three daily fractions of five Gy from day 10 to 12 (Figure 2B).

Tumors in the WT group continued to grow rapidly after inoculation and the tumor volume on day 20 was (1145.0 ± 321.7) mm^3^. (Figure 2C). Tumor growth in the WT-LRT group slowed down after LRT, but continued to grow thereafter and the tumor volume on day 20 was 370.5 ± 83.1 mm^3^ (Figure 2D). Tumors in the IRF3KO group grew rapidly in the early phase after inoculation, but began to shrink on day 13 without treatment and the tumor volume on day 20 was (214.0 ± 55.7) mm^3^ (Figure 2E). Tumors in the IRF3KO-LRT group also grew rapidly in the early phase, but shrank rapidly after LRT and became very small fragments on day 20 and the tumor volume was (79.3 ± 31.1) mm^3^ (Figure 2F,G). Figure 2H shows the tumor growth curves in all groups. The tumor volumes of WT and IRF3KO mice on day 10 before LRT were (260.6 ± 37.6) mm^3^ and (216.1 ± 27.5) mm^3^, respectively, and the tumors in IRF3KO mice were significantly larger than those in WT mice before starting radiation (*p* = 0.01) (Figure 2I). However, the tumor volume was reversed thereafter, and on day 20 tumors in IRF3KO group were significantly smaller than those in WT group (*p* = 0.004). On day 20, the tumors in the WT-LRT group were significantly smaller than the tumors in the WT group (*p* = 0.002). Similarly, in IRF3KO mice, the tumors in the IRF3KO-LRT group were significantly smaller than the tumors in the IRF3KO group (*p* = 0.003). Moreover, the tumors in the IRF3KO-LRT group were significantly smaller than those in the WT-LRT group (*p* < 0.001).

### 3.3. Immune Cell Infiltration in Tumor Microenvironment with LRT in WT vs. IRF3KO Mice

**Figure 3:**
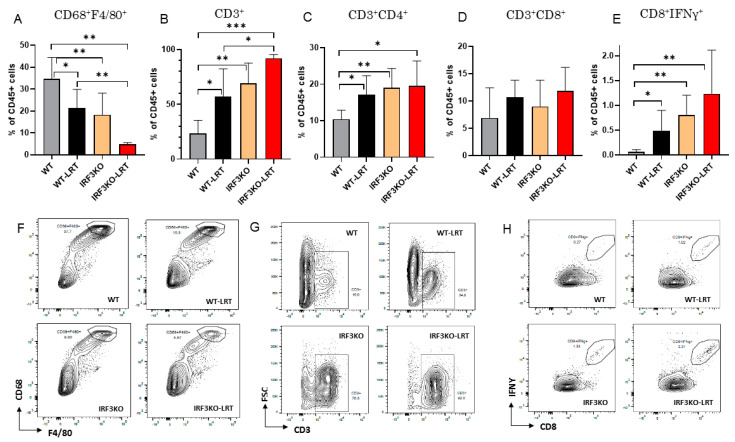
Flow cytometry analysis of immune cells infiltrated into AE17 tumors on day 20. (**A**,**F**) CD68+F4/80+ cells, (**B**,**G**) CD3+ cells, (**C**) CD3+CD4+ cells, (**D**) CD3+CD8+ cells, (**E**,**H**) CD8+IFNγ+ cells in the tumors; data are mean ± SEM (WT, WT-LRT, IRF3KO: *n* = 5 and IRF3KO-LRT: *n* = 3 per group). *, *p* < 0.05; **, *p* < 0.01; ***, *p* < 0.001, unpaired *t* test.

Immune cells infiltrating the tumor on day 20 were analyzed using flow cytometry. The tumor on day 20 in the IRF3KO-LRT group was too small to obtain sufficient cells from the tumors for flow cytometry analysis in two mice, thus three mice were analyzed. Macrophages (CD68^+^F4/80^+^) infiltrating the tumor were significantly reduced in tumors of IRF3KO and IRF3KO-LRT group compared to WT group (*p* = 0.031 and 0.002, respectively) (Figure 3A,F). On the other hand, T lymphocytes (CD3^+^) were significantly increased in tumors of WT-LRT, IRF3KO and IRF3KO-LRT groups compared to WT group (*p* = 0.04, 0.003 and <0.001, respectively) (Figure 3B,G). According to the T lymphocytes, CD4^+^ T lymphocytes (CD3^+^CD4^+^) were significantly increased in the WT-LRT group and the IRF3KO group compared to the WT group (*p* = 0.042 and 0.014 respectively) (Figure 3C). Although CD8^+^ T lymphocytes (CD3^+^CD8^+^) were not significantly different between the four groups (Figure 3D), CD8^+^IFNγ^+^ T lymphocytes were significantly increased in the IRF3KO group compared to the WT group (*p* = 0.029) (Figure 3E,H).

### 3.4. IRF3 and Key Genes in the cGAS/STING Signaling Pathway Associated with Prognosis in TCGA MESO Cohort

**Figure 4:**
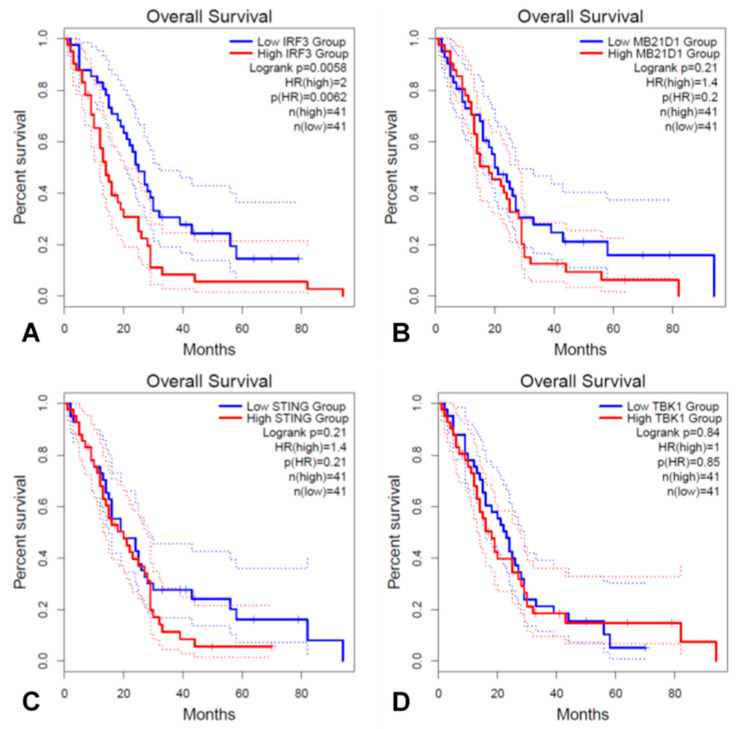
Survival analysis based on mRNA expression of four key genes in cGAS/STING signaling pathway in MESO cohort from TCGA data. (**A**) IRF3, (**B**) MB21D1, (**C**) TMEM173 (STING), and (**D**) TBK1. Kaplan–Meier survival curves were plotted and shown with the HR, a 95% CI, and a log rank test *p* value.

We carried out a Kaplan–Meier analysis of OS using GEPIA2 to determine whether the four key genes of the cGAS/STING signaling pathway are associated with the prognosis of MPM patients. The four genes analyzed are *MB21D1* (encoding cGAS), *TMEM173* (encoding STING1), *TBK1* and *IRF3*. Survival curves were plotted based on the expression level of the four genes associated with overall survival (Figure 4). The results of in silico analysis showed that high expression of *IRF3* had significantly poorer OS than the low expression group (*p* = 0.0058) (Figure 4A), whereas other gene expression of *MB21D1* (Figure 4B), *TMEM173 (STING)* (Figure 4C), and *TBK1* (Figure 4D) were not associated with prognosis.

### 3.5. Correlation Analysis of IRF3 Expression with the Immune Checkpoints and Comparison of Epithelioid vs. Non-Epithelioid Subtype in TCGA MESO

**Figure 5:**
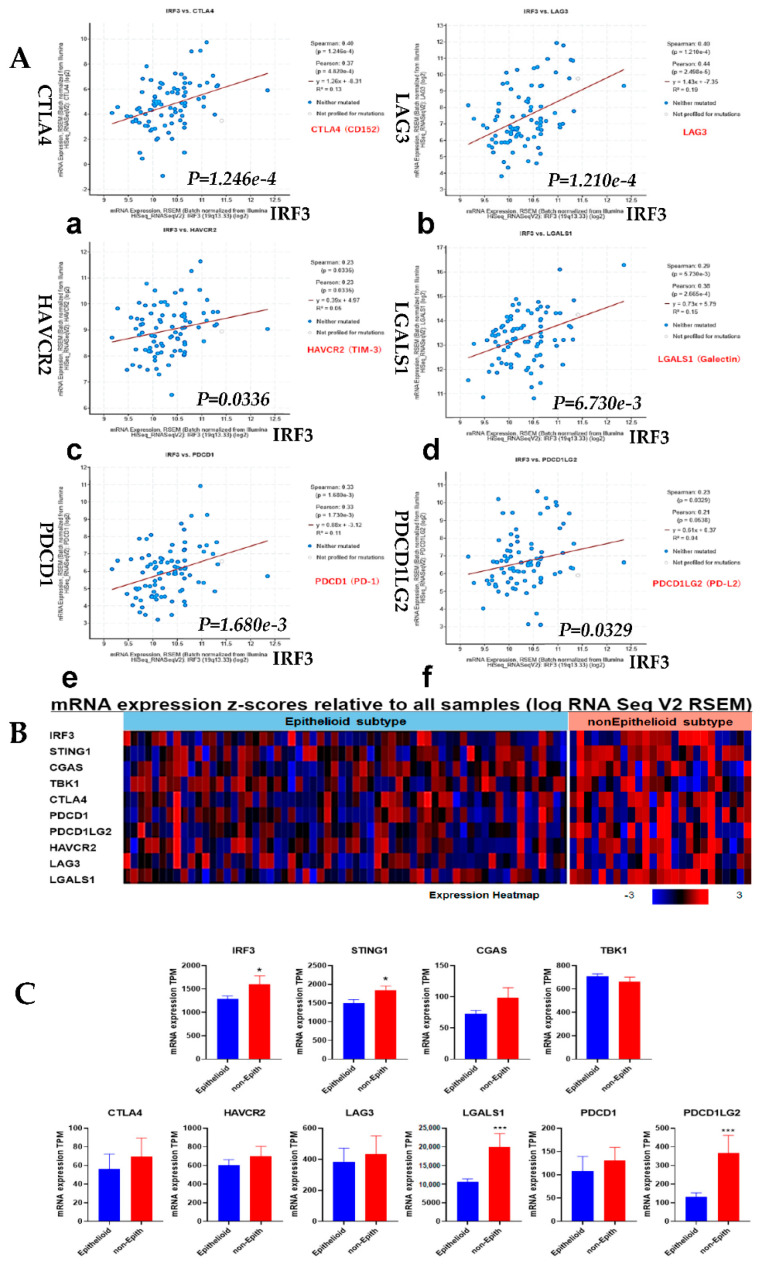
Correlation analysis of IRF3 expression with the immune checkpoints in TCGA MESO. (**A**). *IRF3* gene expression correlated with the immune checkpoint genes expression: (**a**) CTLA4 (CD152); (**b**) LAG3; (**c**) HAVCR2 (TIM-3); (**d**) LGALS1 (Galectin); (**e**) PDCD1 (PD-1); (**f**) PDCD1LG2 (PD-L2). The correlation curves were generated automatically by the online program when importing the gene name of interest. The name of both axes is mRNA expression, RSEM (batch normalized from Illumina HiSeq_RNASeqV2). For all X axes, the name is IRF3, the difference is Y axis as shown by gene names. (**B**). Heat map of mRNA expression of cGAS/STING signaling pathway and immune checkpoint genes in epithelioid vs. non-epithelioid subtypes of MESO. (**C**). Quantification of cGAS/STING signaling pathway and immune checkpoint genes in epithelioid vs. non-epithelioid subtypes of MESO. *, *p* < 0.05; ***, *p* < 0.001, unpaired *t* test.

Correlation analysis of gene expression of *IRF3* with the immune checkpoints in TCGA MESO showed significant correlation (Figure 5A). The mRNA expression of *IRF3* and genes of the cGAS/STING signaling pathway and immune checkpoints in Z score were shown in heatmap and quantification in transcript per million (TPM) of each gene, to compare the difference between epithelioid and non-epithelioid subtype (Figure 5B,C). The expression of *IRF3* and genes of the cGAS/STING signaling pathway was significantly higher in the non-epithelioid subtype than that in the epithelioid subtype (Figure 5B). On the other hand, the expression of immune checkpoint genes tended to be more elevated in the non-epithelioid subtype. Genes *LGALS1* (Galectin) and *PDCD1LG2* (PD-L2) were more significantly expressed in the non-epithelioid subtype compared to the epithelioid subtype (Figure 5C).

Gene expression of *IRF3* and the cGAS/STING signaling pathway and immune checkpoints in our scRNA-Seq data from MESO patients.

**Figure 6:**
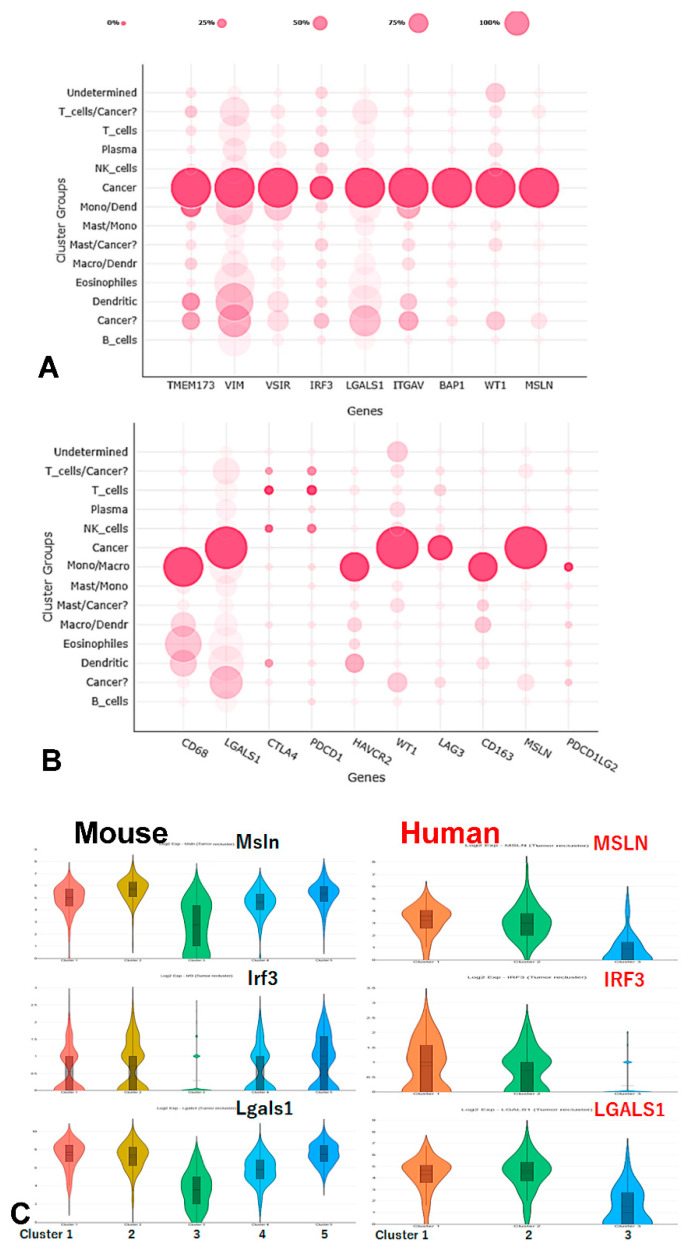
Gene expression of IRF3 and the cGAS/STING signaling pathway and immune checkpoints in our scRNA-Seq data from MESO patients. (**A**) Dotplot of IRF3 and cGAS/STING signaling pathway genes; (**B**) Dotplot of IRF3 and the immune checkpoint genes. Monocyte/macrophage markers (CD68/CD163) and mesothelioma markers (MSLN/WT1) were used as references to identify where these genes were expressed. (**C**) IRF3 and LGALS1 are expressed in high levels in both murine and human mesothelioma cells from scRNA-Seq data.

Dotplot of *IRF3* and the cGAS/STING signaling pathway genes showed that *IRF3* and *STING* (*TMEM173)* were expressed in mesothelioma tumor cells, while *TBK1* and *CGAS* expressed mainly in monocyte/macrophages (Figure 6A). IRF3 expression and the immune checkpoint genes significantly correlated with *LAG3* and *LGALS1* (Galectin) genes expression in mesothelioma cells, while positive correlation was found between *IRF3* and *HAVCR2* (TIM-3) or *PDCD1LG2* (PD-L2) expression in monocytes/macrophages (Figure 6B). Strikingly, *IRF3* and *LGALS1* (Galectin-1) genes were highly expressed in both murine and human mesothelioma cells (Figure 6C).

## 4. Discussion

Our results indicate that *IRF3* may play critical roles in the immunosuppressive microenvironment of mesothelioma. After knocking out this gene, tumor growth was delayed or even completely rejected in some models, while the tumors in WT mice always continued to grow.

A previous report analyzed the relationship between the mRNA expression of key molecules of the cGAS/STING signaling pathway and OS in various cancer using TCGA data [30]. In this study, high expression of *MB21D1* was associated with poor prognosis in colorectal carcinoma, and high expression of *TBK1* was associated with poor prognosis in lung adenocarcinoma and good prognosis in rectal adenocarcinoma. High expression of *IRF3* was associated with poor prognosis in colorectal carcinoma, kidney renal clear cell carcinoma, and prostate adenocarcinoma, but with good prognosis in pancreatic adenocarcinoma, suggesting that the cGAS/STING signaling pathway plays an organ-specific role in tumor progression. In our analysis using TCGA data, the expression of *MB21D1*, *TMEM173*, and *TBK1* was not associated with prognosis, whereas high expression of *IRF3* correlated with poor OS in MESO patients, indicating that *IRF3* is strongly involved in tumor progression in MESO. The cGAS/STING signaling pathway is generally thought to mitigate tumorigenesis, and administration of *STING* agonists has been shown to significantly inhibit tumor development in animal studies via diverse mechanisms [31]. However, some studies have also reported that activation of *STING* promotes the development of certain types of cancers [32,33]. Duguay, et al. reported that melanoma cells transfected with *IRF3* inhibited tumor development by recruiting inflammatory cells to the site of the tumor [34]. Moreover, it has been reported that ectopic expression of *IRF3* inhibited cell growth by blocking DNA synthesis and inducing apoptosis [35]. On the other hand, Yanai, et al. demonstrated that melanoma tumor growth in IRF3KO mice was significantly suppressed compared to WT mice [36].

Interestingly, the tumor infiltrating macrophages of IRF3KO mice on day 20 were significantly reduced compared to those of WT mice. It has been reported that the STING signaling pathway was associated with monocyte migration [37]. Moreover, Liu et al. demonstrated that depletion of IRF3 attenuated adhesion molecule secretion and subsequent infiltration of macrophages into atherosclerotic lesion [38]. Tumor associated macrophages (TAMs) are known to correlate with the prognosis of various cancers [39], and inhibitors of signaling pathways mediated by macrophage colony stimulating factor (M-CSF), which is important for differentiation and migration of TAMs, are promising immunotherapies [40]. Numerous studies have demonstrated that TAMs could suppress naïve T cell proliferation in vitro, suggesting that macrophages can directly suppress T cell function [41,42].

In our study, tumor-infiltrating T lymphocytes, especially CD4^+^ T lymphocytes, were significantly increased in IRF3KO mice compared to WT mice. No significant change in total CD8^+^ T cells was observed in the tumor microenvironment, but CD8^+^IFNγ^+^ T cells were observed to significantly increase in IRF3KO mice, suggesting that macrophages downregulated the presence of tumor infiltrating CD8^+^IFNγ^+^ T cells.

Tumor growth was significantly suppressed in the WT mice with LRT compared with the group without LRT, as we previously reported [25]. Tumor growth was significantly suppressed in the IRF3KO mice after LRT as compared with the group without LRT. The tumor volume of IRF3KO mice with LRT was significantly smaller than that of WT mice with LRT, indicating a synergistic effect of radiation and IRF3KO. Chen, et al. reported that abolishing type I IFN signaling by knocking out interferon α/β receptor subunit 1 2(*IFNAR1*) provoked a pronounced immune response after tumor ionizing radiation from four murine cancer cell lines, including mesothelioma. This enhanced response was CD8^+^ T cell dependent and mediated by increased susceptibility to T cell-mediated killing [43].

Major findings in this study indicate that total macrophages infiltrating the tumor were significantly reduced in tumors of IRF3KO and IRF3KO-LRT groups compared to WT group, while T cells, especially CD4 T cells were significantly increased in tumors of WT-LRT, IRF3KO and IRF3KO-LRT groups compared to WT group. Local radiation can impact the immune system as demonstrated by the abscopal effect, which is able to drive the regression and rejection of non-irradiated, distant tumor lesions [44,45]. In a recent study analyzing the role of IRF3 in inflammation and immunity by conditional and specifically targeted gene ablation in mice, Corales, et al. found that immune cell ontogeny and frequencies of immune cell types were unaffected when IRF3 was selectively inactivated in either T cells or B cells in the mice [32]. Supporting this observation, recent evidence has also shown that IRF3 acts principally within the stromal compartment to drive the networks TNF-α, IL-1β, and IL-6 in WT mice, whereas MCP-1 and IL-6 predominated in IRF3-KO mice. These studies show that IRF3KO affects macrophages by changing their capacity to produce IL-6 and MCP-1 [46]. Their results could explain our findings.

The results shown in Figure 4; Figure 5 are an analysis of IRF3 expression in tumor cells, which differ from IRF3 expression in normal cells but support the important role of IRF3 in mesothelioma progression. However, it is difficult to evaluate the expression of specific factors in normal mesothelial cells in the database, as IRF3 has low expression level in most tissues and low specificity in cell types.

Together, IRF3KO triggered an anti-tumor immune response leading to tumor rejection. Correlation analysis of *IRF3* expression in TCGA MESO showed that *IRF3* positively correlated with the immune checkpoints *CTLA4*, *LAG3*, *HAVCR2* (TIM-3), *LGALS1* (Galectin) and *PDCD1* (PD-1)/*PDCD1LG2* (PD-L2). The mRNA expression of cGAS/STIG signaling pathway and immune checkpoint genes in epithelioid *vs.* non-epithelioid subtypes showed that *LGALS1* is one of the most outstanding genes.

T cell immunoglobulin and mucin-containing molecule 3 (TIM-3) is known as an important immune checkpoint. TIM-3 blockade is being investigated in a wide variety of cancers. Expression of TIM-3 on CD8^+^ T cells in the tumor microenvironment is considered a cardinal sign of T cell dysfunction, therefore, TIM-3 blockade could promote anti-tumor immunity by regulating inflammasome activation [47]. TIM-3 upregulation and regulatory T cell infiltration have shown to be able to mediate the resistance to radiotherapy and PD-L1 blockade [48].

Tumor growth is accompanied by immune escape, which is facilitated by immune checkpoint molecules, such as CTLA-4, PD-1/PD-L1/2, and LAG3. However, the role of tumor glycosylation in immune evasion was mostly overlooked before. Galectin-1 (*LGALS1*)/TIM3, a novel immune checkpoint, is a 135 amino acid, 14 kDa, pleiotropic, non-glycosylated, monomeric or homodimeric carbohydrate-binding protein of the prototype galectin family. Targeting glycans could offer new therapeutic opportunities to unleash the blockade of immune checkpoints. Glycosylation of tumor proteins may generate neo-antigens that can serve as novel targets for tumor-specific T cells [49]. The role of glycosylation in tumor microenvironment, tumor progression and metastasis has been associated with glycans and glycan-binding proteins in tumor immunomodulation [50].

**Figure 7:**
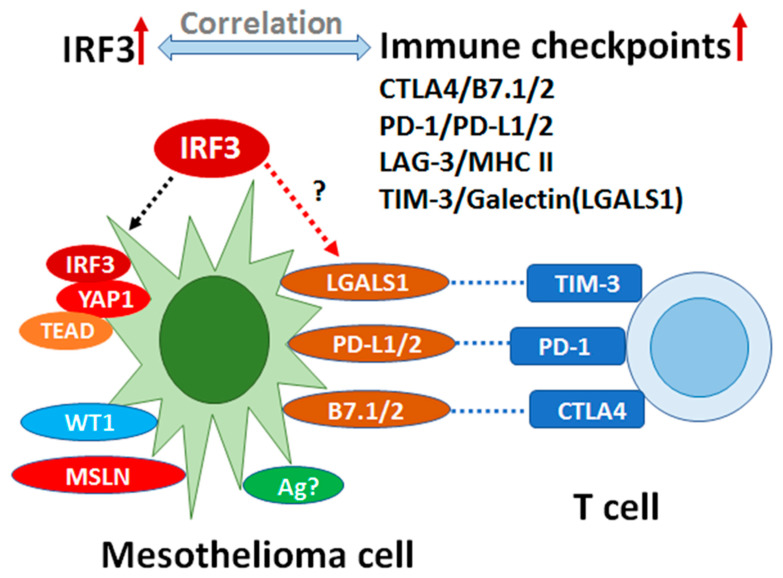
IRF3 may impact mesothelioma cell proliferation as an immunosuppressive transcription factor. Mesothelin (MSLN), Wilm’s tumor protein WT1 and YAP1 are well-known tumor antigens in mesothelioma cells. These molecules have been applied as therapeutic targets and prognostic indicators. Targeting IRF3 was shown to be a YAP agonist target against cancer. In this study, IRF3 knockout resulted in tumor rejection partially or completely, suggesting that IRF3 may play critical roles in immunosuppression. Positive correlation of IRF3 with the immune checkpoints in TCGA MESO and coexpression with LGALS1 in scRNA-Seq data from our patients imply that IRF3 may be an immunosuppressive transcription factor in mesothelioma. Together, IRF3 may be a novel therapeutic target and prognostic marker in MESO.

Mesothelin (MSLN), Wilm’s tumor protein WT1 and YAP1, which are well-known tumor markers in mesothelioma cells, have been applied as therapeutic targets and prognostic indicators. In this study, *IRF3* knockout resulted in tumor rejection partially or completely, suggesting that *IRF3* may play a critical role in immunosuppression. Correlation of *IRF3* with the immune checkpoints in TCGA MESO and coexpression with *LGALS1* in scRNA-Seq data from our patient samples imply that *IRF3* may be an inhibitory transcription regulator in mesothelioma (Figure 7). Targeting *IRF3*, which was shown to be a *YAP* agonist target against gastric cancer, may work similarly in MESO [51]. Overexpression of LGALS1 (Galectin-1) suggests that targeting galectin-1 may inhibit cancer progression by modulating immunosuppressive microenvironment [52]. Galectin-1 can foster an immunosuppressive microenvironment by reprogramming CD8^+^ regulatory T cells and promoting immunotherapy resistance [53,54]. However, other studies have presented different findings demonstrating that IRF3 may be protective against colonic tumorigenesis in particular [55].

There are still several limitations in this study. First, the immune cell populations infiltrating into the tumor microenvironment and contributing to the development of mesothelioma in IRF3KO vs. WT mouse models remains to be delineated. Secondly, it is unclear what effects IRF3KO may have on the number and properties of immune cells and cytokines in those mice with tumor rejection. Finally, the four cell lines used in the experiment were not characterized by major genetic mutations or expression levels of IRF3-related signaling factors and immune-related factors. Further investigations by our group and others will therefore be important to determine the mechanism of IRF3.

## 5. Conclusions

In conclusion, our study indicates that *IRF3* may play an important role in MESO and could be a potential therapeutic target to design novel immunotherapy. *IRF3*, genes of the cGAS/STING signaling pathway and immune checkpoints may provide novel prognostic indicators. Targeting specific genes in this pathway in combination with immune checkpoints could offer new therapeutic opportunities to remove immune blockade. Glycosylation of tumor proteins, *LGALS1* for example, may generate neo-antigens that can serve as novel targets to enhance specific T cell immunity against tumor. *IRF3* may be used as potential targets for therapeutics and prognosis in mesothelioma.

## Data Availability

The data presented in this study are available upon request from Dr. Marc de Perrot Team, due to restrictions eg privacy or ethical. The data are not publicly available at present because the data we presented here are part of an ongoing clinical trial.
